# Do Chemically Modified Oligonucleotide Therapeutics Require Environmental Risk Assessment?

**DOI:** 10.1021/acs.est.6c05885

**Published:** 2026-08-05

**Authors:** Thomas B. Hofstetter, Inga Scheidat, Cresten Mansfeldt, Serina L. Robinson, Michael Sander, Helmut Bürgmann

**Affiliations:** † Eawag, 28499 Swiss Federal Institute of Aquatic Science and Technology, 8600 Dübendorf, Switzerland; ‡ Institute of Biogeochemistry and Pollutant Dynamics (IBP), ETH Zürich, 8092 Zürich, Switzerland; § University of Colorado Boulder, Department of Civil, Environmental, and Architectural Engineering and Environmental Engineering Program, Boulder, Colorado 80309, United States; ∥ Eawag, Swiss Federal Institute of Aquatic Science and Technology, 6047 Kastanienbaum, Switzerland

**Keywords:** antisense oligonucleotide, gapmer, splice-switching oligonucleotide, siRNA oligonucleotide, aptamer, environmental exposure, (eco)toxicity, wastewater treatment, environmental risk assessment

## Abstract

Synthetic modified oligonucleotides (mONs) are a rapidly evolving class of nucleic acid–based therapeutics with an expected increase in market approval rates. mONs are designed to resist enzymatic degradation in patients, and their biological effects arise from chemical modifications and sequence-dependent phenomena comparable to those of biologically active genetic materials. These properties and the absence of experimental evidence on the occurrence and impact of mONs emitted into aquatic environments raise questions about the need for environmental risk assessment (ERA). Here, we evaluate (i) the chemical modifications proposed for mONs and found in approved therapeutics, (ii) pharmacological data on excretion to inform emission scenarios, (iii) analytical approaches for quantification of mONs in environmental matrices, (iv) anticipated distribution and transformation processes, and (v) potential (eco)­toxicological effect profiles of mONs. We propose that focusing on chemical modifications allows for a more generalized approach to assessing mON exposure in engineered and natural aquatic systems. By contrast, the evaluation of effects requires case-by-case studies of chemical modifications and nucleotide sequences in the mON products. We suggest to address the need for ERAs by monitoring mONs in wastewater with newly developed analytical methods as well as experimental studies in laboratory model systems of processes that determine mON exposure and effects.

## Graphical abstract

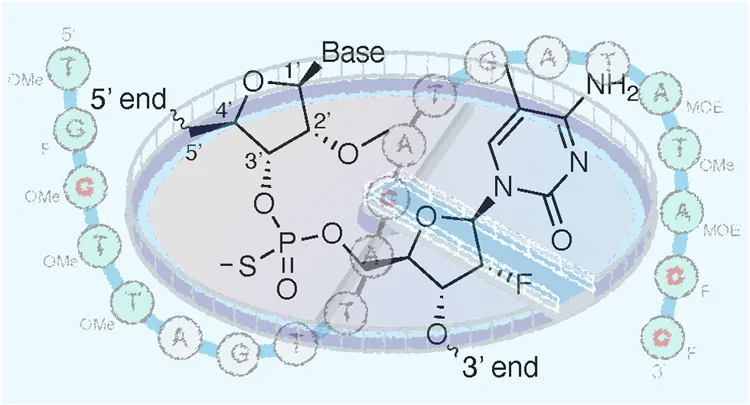

## Introduction

Chemically modified oligonucleotides (mONs) are a rapidly expanding class of nucleic acid–based therapeutics with demonstrated potential to treat diseases long considered undruggable.^1–11^ Twenty-five mONs have been approved as of spring 2026, primarily for treating rare diseases. The approval of a mON for treatment of a widespread cardiovascular disease^12^ and its increasing sales^13^ signal a transition to large-scale use, suggesting a substantial increase in production volumes in the coming years.^14–16^ The mONs’ mode of action relies on their “informational” nature,^3,17^ that is the sequence-specific interaction with targeted RNA through Watson–Crick base pairing or, less frequently, with targeted proteins. mONs act through different biochemical interference strategies primarily for post-transcriptional gene regulation such as targeted nucleic acid degradation and modulation of the spliceosome and other protein activity. The pharmacological performance of the mON, which currently consist of ≈20–60 nucleotides, is largely dictated by extensive chemical modifications which includes all major nucleotide components, namely, the phosphodiester, the ribose unit, and the nucleobases. By improving uptake, tissue targeting, and resistance to nuclease degradation, these modifications are central to clinical application.^2,18,19^ Yet, these chemical modifications may also fundamentally affect mON behavior outside the human body.

Effects of chemical modifications on the absorption, distribution, metabolism, and excretion (ADME) characteristics of mONs in patients are well studied.^20–22^ By contrast, their environmental fate and effects following excretion remain unexplored. Intact and transformed mONs (including fragments) are excreted within urine and feces^23^ into wastewater treatment plants (WWTPs) from where they can enter the aquatic environment. Additional inputs may arise from mON production sites. Despite these anticipated emissions, no experimental or observational data describe their environmental distribution, transformation, or potential (eco)­toxicological effects. While an assessment of environmental behavior and potential risk is mandatory for “conventional” gene therapeutics based on unmodified ONs,^24^ no comparable requirements currently exist for mON therapeutics.

Several considerations argue for systematic investigation of the environmental fate and effects of mONs and a discussion of potential environmental regulation. First, mONs are synthetic therapeutic agents and may therefore require fate and hazard characterization comparable to that of conventional synthetic pharmaceuticals. Second, although mONs are derived from “natural” ONs—which are ubiquitous, chemically labile, and generally considered harmless—the chemical modifications may confer enhanced stability in receiving environments, potentially increasing exposure. Third, selected chemical modifications may increase cytotoxicity,^25^ raising the possibility of adverse effects in nontarget organisms. Fourth, the sequence-specific interactions driving the therapeutic modes of action may also occur in environmental organisms. Fifth, certain regulatory frameworks may broadly consider mONs as biologically active genetic materials and therefore regulated together with genetically modified organisms (GMOs) and other gene therapies.^26,27^ In this Perspective, we therefore identify critical knowledge gaps on the occurrence, fate, and possible (eco)­toxicological effects of mONs and their breakdown products in municipal and hospital wastewater and aquatic environments and evaluate the need for proportionate and scientifically grounded environmental risk assessments for this rapidly growing class of therapeutics.

## Composition and Structure of mONs

mONs are categorized by their biological mode of action, which to some extent defines their molecular features. The biological functions are largely described in recent reviews.^2–4,8^ These functions arise from four main mON categories: (1) Antisense oligonucleotides (ASOs), which comprise gapmers and splice-switching oligonucleotides (SSOs), (2) small interfering RNAs (siRNAs), (3) micro-RNAs (miRNAs), and (4) RNA/DNA aptamers. The recent approval of a telomerase inhibitor^28,29^ underscores ongoing research with the potential to develop additional mON categories for therapeutic use. Notably, except for siRNAs, all mONs are currently single-stranded.

A large and increasing number of chemical modifications have been and continue to be proposed for mONs across all categories.^3,8^ The modifications are distinguished by the chemically altered structural unit of the oligonucleotide, namely, the *phosphodiester* backbone, *ribose* sugar, and *nucleobases* (Figure 1a). To capture modifications beyond the classical ON building blocks, modification of the 2′-ribose position and combined modification of the phosphodiester–ribose backbone are considered separately. We list more than 60 distinct chemical modifications to these five structural units that have been explored in the literature in Section S1 (mON database), illustrating the wide design range available for mON therapeutics.^3,8^ Figure 1b further shows schematic representations of such modifications per mON category based on arbitrarily selected examples of approved drugs. Modifications of the *phosphodiester* moiety currently consist mostly of phosphorothioate (PS) substitutions. PS substitution reduces the susceptibility of the mONs to hydrolytic cleavage by nucleases. This modification occurs on each of the phosphodiester units in mON gapmers. By contrast, only six PS modifications are applied in the siRNA drug *Nedosiran*. To date, alterations of the *backbone* are limited to a single modification type, namely, the phosphoridiamidate morpholino oligonucleotide (PMO) moiety. PMO confers nuclease resistance similar to PS modifications (see SSO *Viltolarsen* in Figure 1b). Alterations of the *2*′*-ribose position* change ribose conformations and ultimately modulate the binding affinity and protein interactions of mONs (see siRNA, miRNA, and aptamer in Figure 1b). These modifications include fluorination (2′-F), methoxylation (2′-OMe), and substitution with 2′-*O*-methoxyethyl ethers (2′-MOE). Modifications of *nucleobases*, as shown in the gapmer *Inotersen*, are restricted to 5-methyluridine (5-MU) and 5-methylcytidine (5-MC), which modulate immunoresponse and duplex stability. Finally, although ten structures for *modified ribose sugars* have been proposed, none have yet been applied in approved therapeutics. In principle, these modifications allow fine-tuning the binding affinity toward the complementary nucleotide sequences of the biological target.

**Figure 1:**
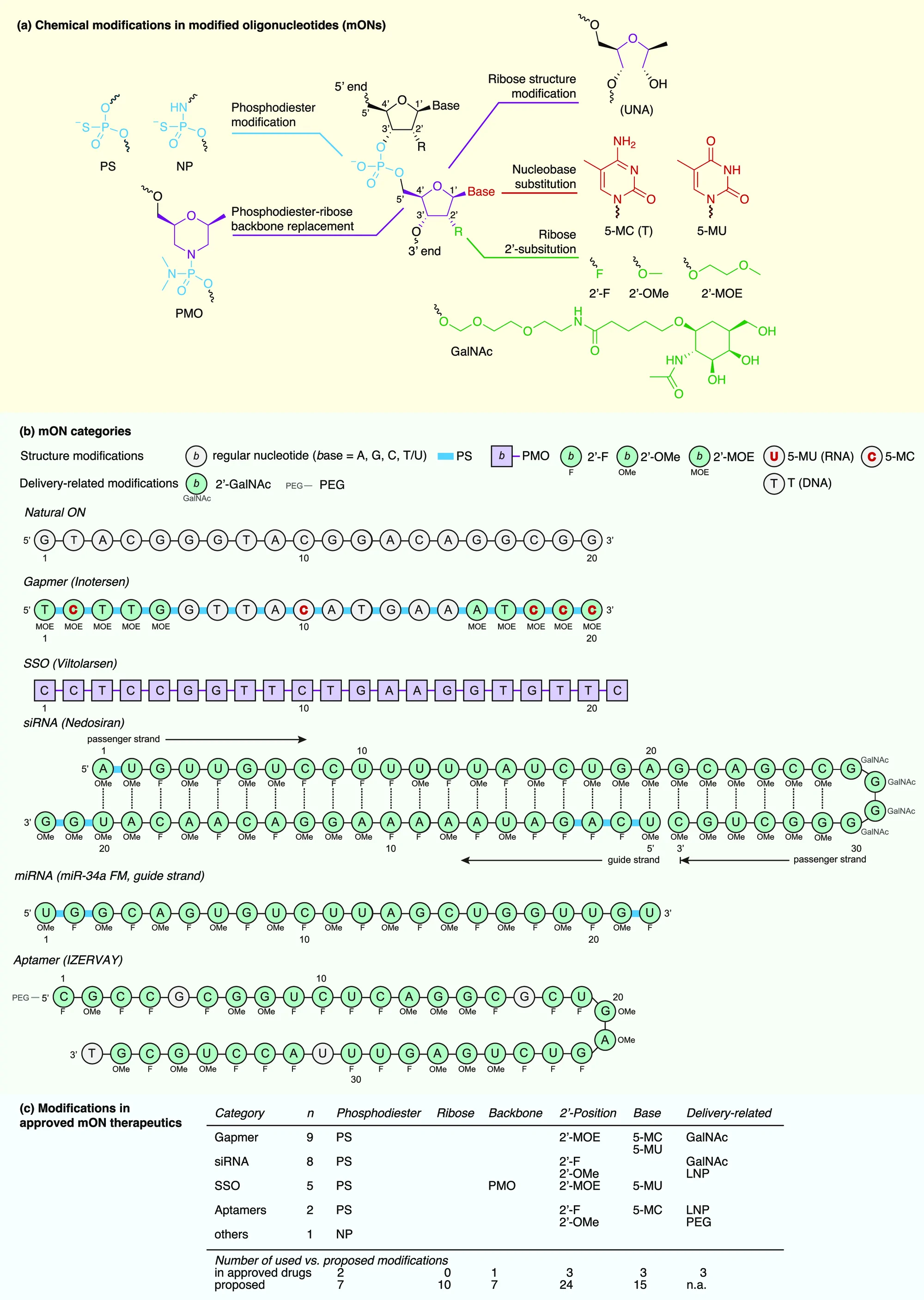
(a) Chemical modifications in approved modified oligonucleotide (mON) therapeutics include the phosphodiester (phosphorothioate, PS, thiophosphoamidate, NP), the phosphodiester-ribose backbone (phosphorodiamidate morpholino oligomer, PMO), substitutions at the 2′-position (2′-fluoro, 2′-F, 2′-*O*-methyl, 2′-OMe, 2′-*O*-methoxyethyl, 2-MOE), and of the nucleobases (5-methyluracil, 5-MU, 5-methylcytosine, 5-MC; note that the 5-MU nucleobase modification in RNA-based mONs corresponds to a thymine nucleobase in a DNA-based mON such as *Inotersen* shown here as example for gapmers). Modifications of the ribose do not exist in approved therapeutics and the structure of unlocked nucleic acid (UNA) is given here for illustration. GalNAc (*N*-acetylgalactosamine) and PEG (polyethylene glycol) stand for delivery-related conjugates. (b) Five functional mON categories with illustration of type and frequency of chemical modifications. The examples shown include *Viltolarsen* (gapmer), *Nedosiran* (siRNA), miR-34a (miRNA in development), and *IZERVAY* (aptamer). (c) Frequency of chemical modifications at different structural units in approved mON therapeutics (as of February 2026) per mON category including delivery-related information (LNP: lipid nanoparticles). Numbers for additional modifications reported in the literature are minimum estimates.

Overall, the 25 approved therapeutics are almost equally divided into RNA- and DNA-based mONs, comprise nine gapmers, eight siRNAs, five SSOs, and two aptamers (no currently approved miRNA-based products) and contain only eight modification types. The table in Figure 1c summarizes the type and frequency of chemical modifications in these approved mON therapeutics. Bulky modifications at the 2′-ribose position and 5′-phosphodiester unit are further introduced to improve mON delivery to target organs and hence therapeutic functionality (Figure 1a/b). Such modifications include covalently bound *N*-acetylgalactosamine (GalNAc), polyethylene glycol (PEG), or antibodies for efficient cellular administration of mONs. Delivery to target organs is also achieved with lipid-based nanoparticles (LNPs) and extracellular vesicles.^2,19,30–33^

## Emission Estimates from Pharmacokinetic Data

Emissions of mONs from patients into wastewater streams can be estimated based on renal and fecal excretion rates generated in pharmacokinetic ADME studies (note that emissions from mON manufacturing could, in principle, also be significant but their evaluation is beyond the scope of this study). The publicly available ADME information covers 19 out of 25 (76%) of the approved mON therapeutics and is compiled in Section S1 for each mON. A summary evaluation of excretion data is provided in Table S1, and preliminary illustrative emission estimates for the currently most widely prescribed siRNA therapeutic, *Inclisiran* (16% excretion rate) are given in Section S2. While emissions are likely negligible for mONs treating rare diseases or with low excretion rates (see below), estimates for *Inclisiran* suggest that annual emissions could approach hundreds of kilograms in the EU. The corresponding concentrations in wastewater may amount to 0.001 μg/L and, in a hypothetical full adoption scenario, could exceed 0.01 μg/L. At the single household level, concentrations may be in the μg/L range. *Inclisiran* represents a maximum emission scenario today. It may, nevertheless, be indicative of increasing levels of emission in the future, as other drugs for treating more widespread diseases (e.g., the hypertension drug *Zilebesiran* ^34,35^ ) are currently in development.

The excretion rates of gapmers and siRNA are generally low (1–27%, Table S1). Numbers for SSOs with PS vs PMO modifications are 0.5% and 27–99%, respectively. Although informative, these data—limited to currently approved mON therapeutics—are insufficient to derive reliable environmental emission estimates broadly across all mONs. Excretion profiles are highly compound- and application-specific. Notably, estimated SSO emissions also correlate with the mode of administration, with direct injection into the cerebrospinal fluid via the spinal canal resulting in substantially lower excretion than intravenous and subcutaneous administration.^23^ These rough estimates highlight the need to establish quantitative relationships between clinical practice—including modes of administration, dosing frequency, and treatment duration—and mON excretion. mON release undoubtedly occurs and may also involve breakdown products relevant in the assessment of risk.

## Monitoring of mON Concentrations in Aqueous Environments

Validated analytical techniques will be essential for determining the emissions of mONs from (bio)­technological production and clinical use and for quantification of mON concentrations in wastewater and aquatic environments. Methods employing nucleotide sequencing and high resolution mass spectrometry for analyses of mONs and their degradation products have been reported,^36–41^ but their application to wastewater matrices requires overcoming several technical constraints. First, the short length and chemical modifications of mONs challenge commonly used environmental molecular biology approaches, such as polymerase chain reaction (PCR) and sequencing-by-synthesis.^42^ The short length of mONs (≤60 nucleotides) limits amplification primer binding, and the chemical modifications can inhibit the required enzymatic activity of polymerases and ligases. Second, the compositional complexity of wastewater matrices challenges analytical approaches routinely applied in pharmacokinetic studies and product development. Low expected concentrations of mONs complicate mass spectrometric analysis, and co-occurring wastewater constituents may inhibit enzyme-linked immunosorbent assays.

Several methodological adaptations partially address these limitations to potentially enable more widespread detection. Stem-loop^43^ or splint ligation^44,45^ modifications to PCR workflows extend the length of the target sequence and remove the interference of chemical modification on primer binding. These modified PCR techniques are attractive in that they reduce interference from chemical modifications and have been demonstrated to detect as few as thousands of copies per reaction (≈10^–17^ g), corresponding to concentrations of approximately 10–100 μg/L in plasma samples, with a recent application achieving detections down to 180 ng/L in mouse plasma and 18–180 ng/g of mouse tissue.^46^ Alternative sequencing technologies, such as nanopore-based sequencing, enable direct detection of both nucleotide sequence and chemical modifications.^47^ Finally, advances in mass spectrometry—including the use of denaturing and nondenaturing chromatographic separation^38^—enable more sensitive characterization and detection of mONs. Reported lower limits of quantification for these approaches typically range from 10 to 100 μg/L (in human and rat plasma).^48^ Notably, a recent study investigating the pharmacokinetics of the RNAi *Vutrisiran* from rat plasma, urine, feces, bile, kidney tissue, and liver tissue demonstrated accurate quantification in the 30–100 μg/L range.^49^ Although these methods are successful when employed for clinical samples, wastewater is a complex assemblage of these biospecimen types and other components. Further method development, validation, and optimization is thus likely required when analyzing wastewater.

The excretion data discussed in Section S2 additionally indicate that pre-concentration steps are likely warranted. Approaches such as solid- or liquid-phase extraction can lower detection limits by factors of 10^2^ to 10^4^, indicating that detection at environmentally relevant concentrations may be feasible. However, wastewater matrix interference may increase effective detection limits, interfere with assay performance, or result in false positives. Similar to the careful design of assays for the recovery of mONs from tissue samples,^50^ assaying wastewater samples will require substantial method validation at environmentally relevant concentrations.

## Exposure Assessment: Distribution and Transformation Processes of mONs

Transport, bioavailability, and effects of mONs are controlled by their dissolved, aqueous concentration. Quantification of dissolved mON fractions for an exposure assessment necessitates an understanding of distribution and (bio)­transformation processes. Due to the lack of empirical observations, the following assessments are qualitatively and based on existing knowledge for natural ONs and by considering the effects of the chemical modifications (Figure 2). In our discussion, we ignore the role of delivery-related GalNAc modifications, LNPs, and vesicles and focus on so-called “naked” mONs. In fact, 10 of the 25 approved mON therapeutics carry GalNAc modifications (Section S1), which are known to be cleaved off the mON in the liver,^2,51,52^ while only 4 are applied in LNP formulations.

**Figure 2:**
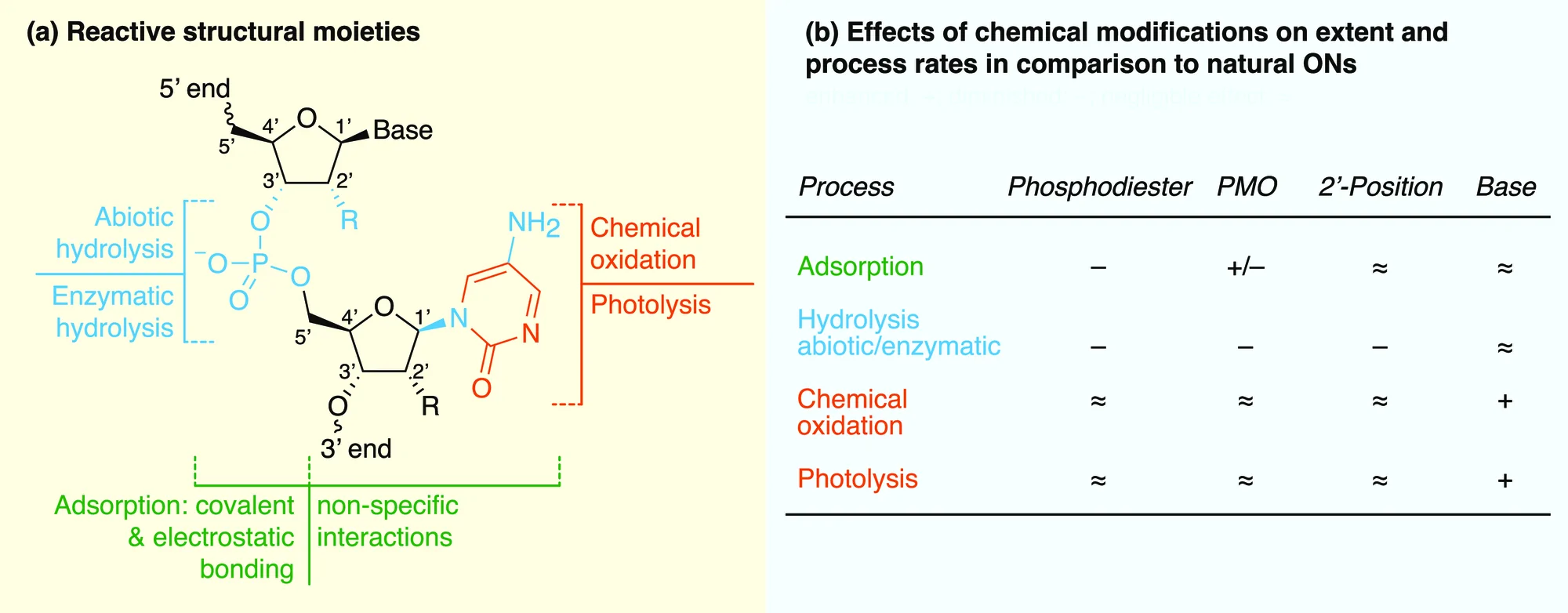
(a) Primary reactive moieties of ONs with relevance for sorption as well as abiotic and enzymatic transformation of mONs illustrated exemplarily in a cytosine nucleotide. (b) Qualitative assessment of the effects of chemical modification on extent of sorption and rates of transformation processes in comparison to natural ONs. Increase (+), reduction (−), both effects (+/–), or no change expected (≈). Note that this qualitative classification of individual processes does not account for the substrate specificity of enzymatic hydrolyses.

### Distribution Processes

Adsorption of mONs to sludge particles within WWTPs will be the main process controlling the distribution of mONs and, thus, their dissolved fraction. This adsorption process is controlled by the type and strength of van der Waals interactions, hydrogen bonding, and electrostatic interactions between the mONs and the particle surfaces (Figure 2a). Phosphodiester groups in mONs can also covalently bind to surfaces of iron minerals formed from Fe­(III) chloride addition including iron phosphates and oxides.

Based on existing ON adsorption studies, electrostatic interactions will likely drive overall mON adsorption. For example, mONs containing the unmodified phosphodiester backbone carry a negative charge and thus will be attracted to positively charged and repulsed from negatively charged particle surfaces.^53,54^ Substitution of phosphate by PS retains the negative charge (Figure 2b) but lowers the charge density of the group and might thus slightly decrease electrostatic attraction to positively charged particles while lowering repulsion from negative adsorbent surfaces. The strength of these electrostatic interactions will, however, be modulated by solution pH (by altering the particles’ net charge) and ionic strength (by attenuating electrostatic interactions). However, the presence of multivalent cations may give rise to cation bridging between mONs and negatively charged surfaces, which may overcome electrostatic repulsion. Notably, common backbone modifications can directly affect the charge and therefore the adsorption of mONs. Electrostatic interactions may also be diminished for mONs with PMO substitution, as this modification removes the negative backbone charge. Whether the diminished ability to undergo electrostatic interaction is compensated by nonspecific, that is dispersive and H-bonding interactions pertinent to uncharged moieties of mONs, is unclear and requires experimental study. Additional base modifications may alter mON adsorption behavior at close contact with sludge floc surfaces, but these effects are likely secondary compared to those modifications that affect mON charge and thus electrostatic interactions.

### Abiotic and Enzymatic Transformations of mONs

Hydrolysis, both abiotic and enzyme-catalyzed, is anticipated to be the key transformation pathway of mONs in WWTPs and aquatic environments, based on insights from ON transformation.^55–58^ Hydrolysis reactions occur primarily at the phosphodiester backbone linkage and, to a smaller extent, at the *N*-glycosidic bond or amine moieties of purine and pyrimidine nucleobases (Figure 2a). Catalysis by dissolved metal ions commonly added in wastewater treatment (e.g., through addition of Fe­(III) salts for phosphate removal) can accelerate hydrolysis,^59–63^ but the relevance of this reaction requires investigation. Further, depending on whether the wastewater treatment contains a fourth treatment stage, chemical oxidation (i.e., by added ozone, chlorine, or chlorine dioxide) and/or ultraviolet light photolysis may further contribute to mON transformation.

#### Abiotic Reactions

Abiotic hydrolysis of phosphodiester bonds in DNA-based mONs, particularly when double-stranded, is too slow (e.g., DNA hydrolysis half-life of approximately 30 million years)^64–67^ to be a relevant transformation pathway. In RNA-based ONs, however, the hydroxyl group in the ribose 2′-position is known to act as an intramolecular nucleophile, increasing hydrolysis rate constants by orders of magnitude and resulting in half-lives between 8 and 80 days at circumneutral pH.^68–70^ Abiotic phosphodiester hydrolysis may thus be a relevant transformation pathway for RNA-based mONs in wastewater. Substitution of the 2′-hydroxyl group in RNA-based mONs with 2′-F, 2′-OMe, or 2′-MOE are expected to substantially lower the abiotic phosphodiester hydrolysis rates as 2′-modifications inhibit the mechanism pertinent to intramolecular RNA hydrolysis.^68^ The same is expected for PS modifications.^71^ Similar conclusions apply to PMO-mONs, although investigations into their stability have primarily focused on enzymatic hydrolysis.

Hydrolysis of *N*-glycosidic bonds are favored in DNA-based mONs but are even slower than the hydrolysis of the phosphodiester backbone at neutral pH.^72^ mONs with substitutions in the 2′-position exhibit even lower reactivity and make this transformation unlikely during wastewater treatment. Similarly, hydrolytic deamination of nucleobases, namely, adenosine, guanine, and cytosine, show half-lives of months to years and are thus expected to be negligible.

Oxidative and photolytic transformations of mONs will involve the nitrogen-containing moieties of nucleobases rather than the backbone (Figure 2), although radical intermediates of such processes can attack other structural units in the mONs. Oxidative and photolytic water treatment is thus expected to increase mON transformation and thus decrease their release rates into receiving waters. Chlorine (as HOCl and OCl^–^) reacts with exocyclic amine moieties of cytosine, adenine, and guanine as well as with heterocyclic NH-groups.^73,74^ Oxidation by chlorine dioxide, in contrast, selectively targets guanine.^75,76^ Reactions of ozone with nucleobases proceed via cycloaddition mechanisms, yielding a range of oxidatively modified and ring-cleavage products.^77–80^ Because nucleobases absorb ultraviolet light, mONs are expected to undergo direct photolysis, with pyrimidines generally being more reactive than purines.^8,81–85^ Moreover, photolysis rates are predicted to be higher for single than double-stranded and higher for DNA- vs RNA-based mONs.^86^ Methylation of nucleobases, as in 5-MC and 5-MU modifications in mONs, slightly increases the electron-density of these moieties and may enhance susceptibility to electrophilic attack by oxidants and in photolysis. The magnitude of this effect is presumably small and primarily relevant for single-stranded mONs.

#### Enzyme-Catalyzed Transformations

Enzymatic hydrolysis is well established as a major transformation pathway of ONs.^55^ Rates of these reactions are expected to be lower for mONs modified in the phosphodiester linkages, backbones, and 2′-positions. Fully PMO-modified SSOs (Figure 1b) completely inhibited the activity of 13 tested hydrolases.^87,88^ By contrast, PS modification slowed transformation only if present at both mON termini.^89^ Modifications at the 2′-positions alter mON structure and can thereby inhibit enzymatic access and activity.^90^ Given that enzyme cascades are also used for mON synthesis,^15,16^ enzymatic transformation of mONs is generally possible. The principal challenge is to assess whether the receiving environment contains enzymes that recognize mONs as substrates. This assessment requires comprehensive characterization of the enzyme pool and their substrate specificity.

Hydrolysis of phosphodiester bonds in the presence of Mg^2+^, Zn^2+^, and Ni^2+^ cofactors is catalyzed by hydrolases and lyases,^91–93^ collectively referred to as nucleases. Nucleases can accelerate rates of hydrolysis by up to 16 orders of magnitude.^94^ Additionally, nucleotidases catalyze downstream dephosphorylation to nucleosides^95^ and may contribute to mON transformation. Nucleases are distinguished by their specificity toward RNA and DNA—acting on both or selectively on either RNA or DNA^96^—and by their mode of action as exo- or endonucleases, which determines the breakdown pathway and length distribution of transformation products. Nucleases are widely distributed in wastewater and aquatic environments^97,98^ and are therefore likely to contribute to mON transformation, although direct experimental evidence remains scarce. Howard et al.^99^ indirectly demonstrated enzyme-mediated transformation of mONs by documenting slower degradation of PS- and 2′-F-modified siRNA pesticide in experiments with agricultural soil, insect saliva, and gut nucleases, further illustrating that the substrate specificity of nucleases also extends to chemical modifications. This specificity was highlighted by the CRISPR-Cas endo­(deoxy)­ribonucleases being inhibited by PS and 2′-F modifications, but not by 2′-OMe modifications.^100,101^ The methylation of nucleobases (5-MC and 5-MU) can further trigger the activity of desoxyribonucleases following the process for DNA-alkylation damage repair.^102^

In summary, adsorption as well as chemical and enzymatic transformation processes of mONs are expected to play a central role in the exposure assessment, in that they can both lower and increase their aqueous concentrations and change their molecular configuration in the environment. We posit that rates of these processes will vary considerably between mONs and may be determined by the type and frequency of chemical modifications.

## (Eco)toxicological Effects of mONs

Since empirical or experimental evidence from environmental systems is not available, an evaluation of the potential adverse effects of mONs in the environment currently has to rely on extrapolations of pharmacological evidence from human cell lines, animal models, and clinical trials. Reported toxicities are currently restricted to standard high-dose trials with concentrations in the μM range (in vitro) and high mg/kg (in vivo). Those cannot be extrapolated to (expected) concentration ranges in wastewater and natural aquatic systems. Comparisons with adverse effects of other, nucleic acid-based compound classes, including GMOs and their DNA, nucleic acids for gene therapy, unmodified ONs, and RNAi pesticides provide further guidance (Table 1). We illustrate in the following that mONs are expected to exhibit a distinct environmental effect profile. For the discussion of adverse effects of mONs, we follow the pharmacological concept of distinguishing sequence-independent, that is chemical modifications-related effects, from sequence-dependent phenomena. Recent work on the design principles for mON therapeutics^103^ suggests, however, that the synergistic interplay of chemical modifications and nucleotide sequence must also be accounted for when assessing the effects of mONs.

**Table 1:** Comparison of Different (Eco)­toxicological Effect Categories of mONs in Comparison to Various Genetically Active or Genetically Engineered Materials. Therapeutic nucleosides refers to nucleoside-based drugs for viral or cancer therapy. “yes”/“no”-statements are generalized assessments, exceptions may exist. Natural DNA refers to unmodified extracellular long-chain natural nucleic acids without additional risk factors. Together with unmodified (short-chain) oligonucleotides, they represent the natural reference that would be exempt from ERA considerations. Therapeutic DNA/RNA are longer chain nucleic acids for medical applications, such as used in gene therapy or RNA vaccines. RNAi pesticides refer to gene silencing RNA-based pesticides with intended lethal effect in targeted pests. RNAi pesticides can largely be considered unmodified medium-sized oligonucleotides. GMO stands for DNA in or from genetically modified organisms (e.g., bacteria, plants). “rare” indicates an effect is possible or has been considered but appears unlikely to be a major concern. If no references are given the classification is assumed to be generally accepted

	effect category	mON and monomers	therapeutic nucleosides	unmodified ON	natural DNA	therapeutic DNA/RNA	RNAi pesticides	GMOs
*sequence-independent effects*								
1	general toxicity	yes^106,108,110^	yes^111,112,127^	no	no	no	no	no
2	mutagenesis by DNA synthesis	yes^122^	yes^111,112,127^	no	rare	no/yes^a^	yes	no
								
*sequence-dependent effects*								
3	nontarget hybridization effects	yes^128,129^	no	no	no	no	yes/rare^121,130^	no/yes^b^
4	toxic effects on nontarget organisms	unknown/rare^109^	no	no	no	no	yes/rare^130^	yes/rare
5	mutagenesis by homologous recombination	yes^126,131^ (enhanced)	no	yes	rare	no	no	no
6	mutagenesis by triple-helix formation	yes/rare^125,132^	no	rare	no	no	no	no
7	functional modification via conjugation	no	no	no	no	no^c^	no	yes (bacteria)
8	functional modification via transduction	no (noncoding)	no	no (noncoding)	no	yes (viral-delivery)	no (noncoding)	yes
9	functional modification via transformation	no (noncoding)	no	no noncoding)	rare	yes	no (noncoding)	yes
10	uncontrolled proliferation	no	no		rare	no	no	yes

a No: mRNA vaccines, nonintegrating gene therapy; yes: therapies with DNA integration or genome editing.

b No: most GMOs; yes: GMOs with pest-control modifications.

c Even when plasmids are used for delivery, they are nonconjugative.

### Sequence-Independent Effects

Effects arising from the chemical modifications are widely reported in the literature on mON development. Modifications such as PS and 2′-F exhibit toxicity^104–107^ when present in oligomers and, for 2′-F, also as monomers, e.g., in possible mON degradation products (Table 1, category 1).^108–110^ Notably, experimental evidence for toxic or genotoxic effects of other modifications is lacking (Table S2). By contrast, chemically modified therapeutic nucleosides, used, for example, in antiviral or cancer therapies, can exhibit a range of well-documented toxic, genotoxic, and immunotoxic effects in cell lines or model organisms.^111,112^ Such drugs are generally assessed under the standard ERA frameworks in the EU^113^ and US^114^ systems because they are non-natural nucleoside analogues, although regulations are case-specific. Modified nucleosides can also induce mutagenesis during DNA synthesis by interactions with polymerases or causing base pair mismatching (Table 1, category 2).^111,112^ However, there is little evidence for this being a concern for nucleosides used in therapeutic mONs (Table S2).

The sequence-independent effects posed by mONs differ substantially from all other unmodified or engineered genetically active materials that are not directly toxic and would generally be considered natural analogues. Drug development processes for mONs have focused on reducing or eliminating the toxic effects of chemical modifications while maintaining or improving the therapeutic benefit or efficacy.^104,115,116^ Approved mON therapeutics have demonstrated acceptable safety profiles for human use, with low toxicity and limited side effects. This optimization is likely to reduce the potential for adverse effects in nontarget organisms in the environment. However, this has not been empirically explored, and there is currently no evidence to rule out increased toxicity of mONs or their constituents toward environmental organisms.

### Sequence-Dependent Effects

#### Nontarget Hybridization

Nontarget hybridization occurs when an mON binds to gene transcripts other than its intended target, potentially leading to specific adverse effects in nontarget functions of cells or organisms (Table 1, category 3).^117,118^ During mON design, nontarget effects are routinely evaluated in human cell lines to mitigate potential side effects. The likelihood of such interactions is generally low given the short length of mONs and thus a limited combinatorial potential, but it cannot be fully ruled out. In addition, hybridization-stabilizing effects of certain chemical modifications in mONs may also affect the likelihood of nontarget interactions.^119^ The substantially greater metagenomic diversity of environmental biota compared with human cells further increases the probability for such interactions. Similar concerns regarding nontarget effects have been raised and studied in more detail for RNA interference (RNAi) pesticides (siRNA) and certain GMOs, e.g., pest-resistant crops.^56,120,121^ Compared to mONs, environmental risks from the latter appear more likely, because these applications are deliberately designed for lethal effect in environmental (micro)­organisms (i.e., the silencing of essential genes in pest organisms). RNAi pesticides are furthermore applied in high doses or are expressed at high levels in RNAi crops (Table 1, categories 3 and 4). However, the risk is still elevated for mONs compared to other genetically active materials, for which it is negligible.

#### Genotoxicity and Mutagenesis

mONs can induce site-directed mutations through mechanisms such as homologous recombination^122,123^ or triple-helix formation,^124^ and modifications can increase this mutagenic potential considerably compared to unmodified ON (Table 1, categories 5 and 6). For research applications, mONs have been specifically designed for the purpose of mutagenesis, and it is reasonable to assume that such interactions could also occur unintentionally in environmental (micro)­organisms. This concern has been considered in drug safety assessments,^125^ and mON therapeutics are therefore subjected to standard assays for mutagenic and genotoxic potential, such as the Ames test, as part of the regulatory approval. Approved mON therapeutics have met regulatory genotoxicity requirements,^125^ whereas research has shown that mONs can, in principle, have such effects, typically at supratherapeutic concentrations (Table S2). However, the Ames test relies on mutagenic effects in a single species (*Salmonella typhimurium*). Because the mutagenic mechanisms considered for mONs are sequence-dependent, the results of such a test may not fully capture risks across the genetic diversity present in the natural ecosystems. Similar to nontarget hybridization by mONs, empirical evidence for the existence or absence of mutagenic effects in such a broader environmental context is currently lacking.

#### Transformation/Gain of Function

While mONs are biologically active genetic material, concerns typically associated with the risk assessment and regulation of GMOs do not apply to them. GMOs may include new or modified genes and may or may not release genetic material. If released, this material may be incorporated into the genome of environmental microorganisms through various mechanisms such as horizontal gene transfer (Table 1, categories 7–9), potentially resulting in heritable traits (gain of function) with unpredictable ecological consequences (category 10). Therapeutic nucleic acid constructs used in gene therapy can encode functions and are specifically designed for genome alteration. Yet, the likelihood of their environmental release in an active configuration is low because of their rapid degradation by nucleases. Gene therapies involving viral vectors are sometimes also considered under GMO-like risk categories, but these types of delivery systems are not used for mONs. Given their short sequences (≈20–60 nucleotides), mONs lack the length and structural features required to efficiently engage in canonical homologous recombination. They also do not carry sufficient genetic information to independently encode heritable functions.^126^ For RNA-based mONs, this risk is even lower, as incorporation would require additional steps, such as reverse transcription. Thus, even in the unlikely case of successful recombination, these events would not result in new expressed traits by environmental (micro)­organisms—the risk posed by GMOs—but would only result in local site-specific mutational effects, as described above (similar to mutagenic chemicals).

## A Case for Environmental Risk Assessment?

Evidence for toxic effects linked to chemical modifications, the lack of experimental data on environmental occurence and fate, and the expected increase of global mON therapeutics production for applications beyond rare diseases call for discussions on the need and procedural approach for the ERA of mON therapeutics. This discussion might become more pressing with the anticipated agricultural use of mONs (e.g., RNAi-mONs for pest control^99^) and their deliberate release into the environment. As a foundation for science-based discussions on the need of ERA for mONs, we propose to address the following knowledge gaps.

First, the environmental exposure of mONs must be quantified. ADME-based emission estimates and systematic monitoring in WWTP can provide realistic emission inventories and should also include industrial mON production facilities. mON measurements appear within reach through extending current analytical methods for advanced nucleotide sequencing and mass spectrometry. The detection of mONs in WWTPs and its effluent would indeed represent important prerequisites for ERAs. Second, processes determining the partitioning and transformation of mONs in aquatic systems require evaluation. mONs are designed to be stable in the bodies of treated patients and therefore have considerable potential to persist in the environment. Laboratory model systems established for studying micropollutant (bio)­degradation allow for assessing how chemical modifications determine partitioning between water, sludge, and sediments, and formation of transformation products such as shorter oligonucleotides or oxidized nucleobases. The outcomes will allow generalized assessments across mON therapeutics based on the types and abundance of chemical modifications, where indications for persistence, exposure, and bioavailability support cases for ERA. Third, specific testing of mON products is required for (eco)­toxicological effects from chemical modifications and nucleotide sequences. Such tests—which should include comparisons with effect categories for GMOs and other nucleic acid–based constructs—currently call for methodological groundwork. Empirical evidence for mON mutagenicity, for example, is limited by in vitro studies using nonclinical concentrations and conditions,^122^ emphasizing the necessity of standardized testing protocols. Bioinformatic screening is already employed for evaluating nontarget effect risks,^119^ and the approach could be expanded to environmental metagenomes for assessing hybridization potential of mONs in nontarget organisms. However, environmental metagenomes are often taxonomically and functionally fragmented, leaving many nonmodel organism genomes incomplete or entirely unrepresented. More complex sequence-dependent interactions are also likely to remain overlooked. Application of bioinformatic screening approaches is thus contingent upon extension of databases and their validation. Collectively, experimental insights into the environmental exposure of mONs, their persistence, and (eco)­toxicological effects will be essential for evaluating the need of ERAs of mONs. Given the combined concerns related to the effects of chemical modification on fate and potential biologic activity of the mONs, we posit that a new framework for ERA of mONs may be warranted.

## Supplementary Material







## Notes

Compilation of chemical modifications used in mONs (mON database) (XLSX)
Summary evaluation of ADME data for mON excretion, emission estimate calculation, (XLSX)
Documentation of mON database and emission estimate calculation, summary evaluation of excretion in % of administered dose in the mON database grouped by mON category, and literature review of the toxicity and genotoxicity of modifications used in approved oligonucleotide therapeutics (PDF)
