# Supplementary material for: Do Chemically Modified Oligonucleotide Therapeutics Require Environmental Risk Assessment?
Source: Environ Sci Technol. 2026 Aug 5;60(32):22174–85. doi: 10.1021/acs.est.6c05885 (PMC13492294; doi:10.1021/acs.est.6c05885)
Supplement: Supplementary file 1 [file es6c05885_si_003.pdf]

Supporting Information

Do Chemically Modified Oligonucleotide Therapeutics Require Environmental Risk Assessment?

Thomas B. Hofstetter[1,2*], Inga Scheidat[1], Cresten Mansfeldt[3], Serina L. Robinson[1,2], Michael Sander[1,2], and Helmut Bürgman[2,4]

[1]Eawag, Swiss Federal Institute of Aquatic Science and Technology, CH-8600 Dübendorf, Switzerland, [2]Institute of Biogeochemistry and Pollutant Dynamics (IBP), ETH Zürich, CH-8092 Zürich, Switzerland; [3]University of Colorado Boulder, Department of Civil, Environmental, and Architectural Engineering and Environmental Engineering Program, Boulder, CO, 80309, USA, [4]Eawag, Swiss Federal Institute of Aquatic Science and Technology, CH-6047 Kastanienbaum, Switzerland

*Corresponding author: thomas.hofstetter@eawag.ch

7 Pages, 2 Tables

Contents

S1 Compilation of chemical modifications in mONs: The mON-database **2**

S2 Summary evaluation of ADME data for mON excretion **3**
S2.1 Illustrative emission estimate calculation 4

S3 Summary of literature survey on toxicity and genotoxicity of chemical modifications in mONs **5**

S1 Compilation of chemical modifications in mONs: The mON-database

The supplementary file "S1_mON_database.xlsx", referred to as "mON-database" in the main manuscript contains the following 5 worksheets.

"mON-therpeutics11 lists the 25 commercially available mON therpeutics (as of February 2026). Entries are listed by their tradenames, active ingredient, mON category, and approval status by the European Medicines Agency (EMA) and US Food and Drug Administration (FDA) . This worksheet organizes mON therapeutics according to their mON category, nucleotide length, type and frequency of chemical modification and includes relevant ADME data.

In total, 25 mON therapeutics were approved by the FDA in the past decades. Note that Formivirsen was retracted from the US-market. Modified oligonucleotides can be qualitatively assessed with regard to their renal and fecal excretion from ADME studies. These data include target organs (e.g., eye, intestine, liver, kidney, skeletal muscle, and CNS), molecular targets, disease, administration route (intrathecal, subcutaneous, vitreal, intravenous) and company name. In addition, treatment doses (in mg for a 70-kg person) are given based on the active substance, dosing intervals (in weeks) and percentages of excreted material were also researched and added. All information on excretion refers to intact, full-length mONs. No data was available on transformation products, including potential secondary metabolites.

"glossary" lists the most important terms used in the list of mON therapeutics.

"modifications" contains 64 chemical modifications reported in the scientific literature for mON therapeutics. Note that this list is not complete as additional modifications are being added. This list is organized according to the chemically modified structure element of the nucleotide (phosphodiester, backbone, ribose-analoga, 2'-substitution of ribose, nucleobase). Modifications are assigned to mON category if this information is available. The sources of information are provided as citation keys.

"citations_literature" contains the bibliographic information corresponding to each citation key including DOI.

"citations_EMA-FDA" is the reference document listing citations of documents provided by EMA, FDA, and Swissmedic for each of the evaluated mON therapeutics.

S2 Summary evaluation of ADME data for mON excretion

Table S1 Summary evaluation of excretion in % of administered dose in the mON database grouped by mON category. Note excretion data is available for 19 out of 25 (76%) of the mONs approved by early 2026. Data in columns designated by the chemical modification represent the average (± standard deviation) number of modification per mON and mON category. n stands for the number of approved mON-therapeutics per category.

**mON-category**	n	**PS**	**PMO**	**2'-F**	**2'-OMe**	**2'-MOE**	**5-MC**	**5-MU**	**Excretion** (%)
Gapmer	8[a]	17 ± 3[b]				10	8 ± 3	5 ± 1	1 - 15
siRNA	6	6 ± 1		13 ± 4	29 ± 9				1 - 27
SSO (PS)	1	17				18	4	7	0.5
SSO (PMO)	4		24 ± 4				8 ± 2	7	27 - 99
Aptamers	1			21	14		1		n.a.[c]

[a] for 1 of 8 gapmers, no data is available for type and frequeny of chemical modifications available;
[b] average number of chemical modification per drug, standard deviations were calculated if a modifications occurs ≥ 3 times; [c] n.a. = not available.

S2.1 Illustrative emission estimate calculation

Current applications of mONs therapeutics target rare diseases, resulting in low overall patient numbers and, correspondingly, emissions into the environment following excretion by patients are expected to be small. This situation is likely to change as mONs are approved for more prevalent conditions as shown here with an illustrative back-of-the-envelope calculation for the siRNA-mON *Inclisiran*. The corresponding data can be found in supplementary file "S2_emission-estimate.xlsx".

Inclisiran is now approved for the treatment of hypercholesterolemia[1] and sales have increased substantially in 2026.[2] In England, nearly 600 000 individuals meet the eligibility criteria for *Inclisiran* treatment although only about 7% of the eligible population have adopted therapy to date.[3,4] The use is expected to increase substantially as long-term clinical trial data for *Inclisiran* become available. Given that high cholesterol affects over 25% of the global population,[5] widespread adoption of *Inclisiran* and similar mON therapeutics (e.g., the hypertension drug *Zilebesiran*[6,7]), could markedly increase mON ond degradation product loads in clinical waste streams and in patient-derived emissions. Therefore, we calculated three illustrative emission scenarios for a single patient household, a hypothetical metropolis, and for England, based on the current adoption rate in England[3,4] and up to a hypothetical maximum (100%). To estimate dilution in wastewater we used reported sewage production values for England or typical planning volumes (150 L/PE/day). Based on these numbers, we also extrapolated potential cumulative emission in the European Union. Approximately 16% of *Inclisiran* is excreted unchanged in urine within the first 48 hours (see mON-database, Section S1). With a yearly dosing regime of approximately 568 mg (but higher in an initial phase of therapy[8]), this would correspond to $\approx$ 94 kg of annual mON emissions to wastewater per one million treated patients.

Locally, in the wastewater of users, average concentrations may exceed 1.5 μg/L. Assuming a hypothetical 50% or higher adoption rate of *Inclisiran*, concentrations near or $\geq$ 0.01 μg/L would be expected in wastewater, while current adoption rates yield concentration levels at or below 0.001 μg/L.

S3 Summary of literature survey on toxicity and genotoxicity of chemical modifications in mONs

Table S2 Summary of the literature review of the toxicity and genotoxicity of modifications used in approved oligonucleotide therapeutics. It should be noted that in many cases, toxicity is strongly influenced by the specific sequence or structure of the ON and the combination with other modifications in the same oligonucleotide.[9] Therefore, the information provided is not necessarily universally applicable.

	**PS**	**PMO**	**2'-F**	**2'-MOE**	**2'-OMe**	**5-MC**	**5-MU**
Oligonucelotide							
Toxicity	Yes[10–12]	Low[13]	Yes[14]	Low[15–17]	Low[18]	NA	No[a]
Genotoxicity	No[11]	No[19]	possible[20]	No[21]	No[15,22]	No[22]	NA
Monomers							
Toxicity	NA	NA	Yes, occ. severe[23]	NA	NA	NA	NA
Genotoxicity	NA	NA	possible[20]	Low[24]	NA	NA	NA

[a] 5-Methylcytidin is a naturally occurring methylated modification and is not considered toxic..[25] NA = not available.

References

[1] Raal, F. J.; Kallend, D.; Ray, K. K.; Turner, T.; Koenig, W.; Wright, R. S.; Wijngaard, P. L.; Curcio, D.; Jaros, M. J.; Leiter, L. A.; Kastelein, J. J. Inclisiran for the treatment of heterozygous familial hypercholesterolemia. *N. Engl. J. Med.* **2020**, *382*, 1520–1530, http://doi.org/10.1056/NEJMoa1913805.

[2] Novartis Investor Presentation, April 28, 2026 "Q1 2026 Results: Leqvio (Inclisiran) Q1 sales grew +69%". page 9, accessed June 20, 2026, https://www.novartis.com/sites/novartis_com/files/q1-2026-investor-presentation.pdf.

[3] Connely, D. Inclisiran prescribed to 7% of expected target population in three years, NHS data reveal. 2026 (accessed 2026-06-22); https://pharmaceutical-journal.com/article/news/inclisiran-prescribed-to-7-of-expected-target-population-in-three-years-nhs-data-reveal.

[4] Fagg, M.; Williams, H. Cardiovascular disease prevention: lipid management including access to inclisiran. 2025 (accessed 2026-06-22); NHS England, https://www.england.nhs.uk/long-read/cvd-disease-prevention-lipid-management-inclisiran/.

[5] Mohamed-Yassin, M.-S.; Baharudin, N.; Abdul-Razak, S.; Ramli, A. S.; Lai, N. M. Global prevalence of dyslipidaemia in adult populations: a systematic review protocol. *BMJ Open* **2021**, *11*, https://doi.org/10.1136/bmjopen-2021-049662.

[6] Verma, S.; Pradhan, A.; Thandi, P. Zilebesiran: an RNA interference agent – its need and potential to transform hypertension treatment. *Egypt. Heart. J.* **2026**, *78*, 12, https://doi.org/10.1186/s43044-026-00724-9.

[7] Desai, A. S.; Webb, D. J.; Taubel, J.; Casey, S.; Cheng, Y.; Robbie, G. J.; Foster, D.; Huang, S. A.; Rhyee, S.; Sweetser, M. T.; Bakris, G. L. Zilebesiran, an RNA interference therapeutic agent for hypertension. *N. Engl. J. Med.* **2023**, *389*, 228–238, http://doi.org/10.1056/NEJMoa2208391.

[8] European Medicines Agency Assessment report Leqvio. 2021 (accessed 2026-06-18); https://www.ema.europa.eu/en/documents/assessment-report/leqvio-epar-public-assessment-report_en.pdf.

[9] Ruan, H.; Dou, D.; Lu, J.; Xiao, X.; Gong, X.; Zhang, X. Off-Target Effects of Oligonucleotides and Approaches of Preclinical Assessments. *SLAS Discovery* **2025**, *35*, 100254, https://doi.org/10.1016/j.slasd.2025.100254.

[10] Crooke, S. T.; Vickers, T. A.; Liang, X.-h. Phosphorothioate modified oligonucleotide–protein interactions. *Nucl. Ac. Res.* **2020**, 5235–5253, https://doi.org/10.1093/nar/gkaa299.

[11] Henry, S. P.; Beattie, G.; Yeh, G.; Chappel, A.; Giclas, P.; Mortari, A.; Jagels, M. A.; Kornbrust, D. J.; Levin, A. A. Complement activation is responsible for acute toxicities in rhesus monkeys treated with a phosphorothioate oligodeoxynucleotide. *Intern. Immunopharm.* **2002**, 1657–1666, https://doi.org/10.1016/S1567-5769(02)00142-X.

[12] Croft, L. V.; Fisher, M.; Barbhuiya, T. K.; El-Kamand, S.; Beard, S.; Rajapakse, A.; Gamsjaeger, R.; Cubeddu, L.; Bolderson, E.; O'Byrne, K.; Richard, D.; Gandhi, N. S. Sequence- and structure-dependent cytotoxicity of phosphorothioate and 2'-O-methyl modified single-stranded oligonucleotides. *Nucleic Acid Therapeutics* **2024**, 143–155, https://doi.org/10.1089/nat.2023.0056.

[13] Carver, M. P.; Charleston, J. S.; Shanks, C.; Zhang, J.; Mense, M.; Sharma, A. K.; Kaur, H.; Sazani, P. Toxicological characterization of exon skipping Phosphorodiamidate morpholino oligomers (PMOs) in non-human primates. *Journal of Neuromuscular Diseases* **2016**, 381–393, https://doi.org/10.3233/JND-160157.

[14] Shen, W.; De Hoyos, C. L.; Sun, H.; Vickers, T. A.; Liang, X.-h.; Crooke, S. T. Acute Hepatotoxicity of 2′ Fluoro-Modified 5–10–5 Gapmer Phosphorothioate Oligonucleotides in Mice Correlates with Intracellular Protein Binding and the Loss of DBHS Proteins. *Nucleic Acids Res.* **2018**, 2204–2217, https://doi.org/10.1093/nar/gky060.

[15] Henry, S. P.; Kim, T.-W.; Kramer-Stickland, K.; Zanardi, T. A.; Fey, R. A.; Levin, A. A. Toxicologic Properties of 2 -O-Methoxyethyl Chimeric Antisense Inhibitors in Animals and Man. *Antisense drug technology: principles, strategies, and applications* **2004**, 327–363, https://doi.org/10.1002/bdrb.20023.

[16] Zanardi, T. A.; Kim, T.-W.; Shen, L.; Serota, D.; Papagiannis, C.; Park, S.-Y.; Kim, Y.; Henry, S. P. Chronic toxicity assessment of 2'-O-Methoxyethyl antisense oligonucleotides in mice. *Nucleic Acid Therapeutics* **2018**, 233–241, https://doi.org/10.1089/nat.2017.0706.

[17] Partridge, W.; Xia, S.; Kwoh, T. J.; Bhanot, S.; Geary, R. S.; Baker, B. F. Improvements in the tolerability profile of 2'-O-Methoxyethyl chimeric antisense oligonucleotides in parallel with advances in design, screening, and other methods. *Nucleic Acid Therapeutics* **2021**, 417–426, https://doi.org/10.1089/nat.2020.0917.

[18] Crooke, S. T.; Baker, B. F.; Kwoh, T. J.; Cheng, W.; Schulz, D. J.; Xia, S.; Salgado, N.; Bui, H.-H.; Hart, C. E.; Burel, S. A.; Younis, H. S.; Geary, R. S.; Henry, S. P.; Bhanot, S. Integrated safety assessment of 2-O-Methoxyethyl chimeric antisense oligonucleotides in non human primates and healthy human volunteers. *Molecular Therapy* **2016**, 1771–1782, https://doi.org/10.1038/mt.2016.136.

[19] Sazani, P.; Weller, D. L.; Shrewsbury, S. B. Safety pharmacology and genotoxicity evaluation of AVI-4658. *International Journal of Toxicology* **2010**, 143–156, https://doi.org/10.1177/1091581809359206.

[20] Richardson, F. C.; Zhang, C.; Lehrman, S. R.; Koc, H.; Swenberg, J. A.; Richardson, K. A.; Bendele, R. A. Quantification of 2'-Fluoro-2'-Deoxyuridine and 2'-Fluoro-2'-Deoxycytidine in DNA and RNA isolated from rats and woodchucks using LC/MS/MS. *Chem. Res. Toxicol.* **2002**, 922–926, https://doi.org/10.1021/tx020014d.

[21] Janas, M. M. et al. Safety evaluation of 2'-Deoxy-2'-Fluoro nucleotides in GalNAc-siRNA conjugates. *Nucleic Acids Res.* **2019**, 3306–3320, https://doi.org/10.1093/nar/gkz140.

[22] Kim, T.-W.; Papagiannis, C. N.; Zwick, L. S.; Snyder, P.; Engelhardt, J. A.; Yu, R. Z.; Hoffmaster, C. M.; Rastogi, A.; Henry, S. P. Carcinogenicity assessment of Inotersen in Tg.rasH2 mice and Sprague-Dawley rats. *Regulatory Toxicology and Pharmacology* **2025**, 105743, https://doi.org/10.1016/j.yrtph.2024.105743.

[23] Pankiewicz, K. W. Fluorinated nucleosides. *Carbohydrate Research* **2000**, 87–105, https://doi.org/10.1021/acs.analchem.4c02667.

[24] Saleh, A. F.; Bachman, M.; Priestley, C. C.; Gooderham, N. J.; Andersson, P.; Henry, S. P.; Edmunds, N. J.; Fellows, M. D. 2'-O-2-Methoxyethyl nucleosides are not phosphorylated or incorporated into the genome of human lymphoblastoid TK6 cells. *Toxicological Sciences* **2018**, 70–78, https://doi.org/10.1093/toxsci/kfy005.

[25] Arguello, A. E.; Li, A.; Sun, X.; Eggert, T. W.; Mairhofer, E.; Kleiner, R. E. Reactivity-dependent profiling of RNA 5-Methylcytidine dioxygenases. *Nature Communications* **2022**, 4176, https://doi.org/10.1038/s41467-022-31876-2.
